# Amperometric bio-sensing of lactate and oxygen concurrently with local field potentials during *status epilepticus*

**DOI:** 10.1016/j.talanta.2023.125302

**Published:** 2023-10-08

**Authors:** Eliana Fernandes, Ana Ledo, Greg A. Gerhardt, Rui M. Barbosa

**Affiliations:** a University of Coimbra, Faculty of Pharmacy, Health Sciences Campus, Azinhaga de Santa Comba, 3000-548, Coimbra, Portugal; b Center for Neuroscience and Cell Biology, University of Coimbra, 3004-504, Coimbra, Portugal; c Center for Microelectrode Technology (CenMeT), Department of Neuroscience, University of Kentucky Medical Center, Lexington, KY, 40536, United States

**Keywords:** Carbon fiber microelectrode, Lactate biosensor, Oxygen sensor, Local field potentials, *In vivo* electrochemistry, *Status epilepticus*

## Abstract

Epilepsy is a prevalent neurological disorder with a complex pathogenesis and unpredictable nature, presenting limited treatment options in >30 % of affected individuals. Neurometabolic abnormalities have been observed in epilepsy patients, suggesting a disruption in the coupling between neural activity and energy metabolism in the brain. In this study, we employed amperometric biosensors based on a modified carbon fiber microelectrode platform to directly and continuously measure lactate and oxygen dynamics in the brain extracellular space. These biosensors demonstrated high sensitivity, selectivity, and rapid response time, enabling *in vivo* measurements with high temporal and spatial resolution. *In vivo* recordings in the cortex of anaesthetized rats revealed rapid and multiphasic fluctuations in extracellular lactate and oxygen levels following neuronal stimulation with high potassium. Furthermore, real-time measurement of lactate and oxygen concentration dynamics concurrently with network electrical activity during *status epilepticus* induced by 4-aminopyridine (4-AP) demonstrated phasic changes in lactate levels that correlated with bursts of electrical activity, while tonic levels of lactate remained stable during seizures. This study highlights the complex interplay between lactate dynamics, electrical activity, and oxygen utilization in epileptic seizures.

## 1. Introduction

Epilepsy is a common neurological disorder characterized by a persistent predisposition to generate seizures due to a sudden and temporary synchronization of neuronal electrical activity [1]. The causes of epilepsy are varied and can include genetic predisposition, traumatic brain injury, brain development disorders, infection, or other conditions [2–4]. Epilepsy can be treated with medication, surgery, and other therapies, but there is currently no cure for epilepsy and its pathogenesis and predictability remain largely unknown. Additionally, despite the constant development of antiepileptic drugs, the percentage of patients with drug refractory epilepsy remains at a high value of >30% [3,5].

Several studies have shown that metabolic abnormalities are common in epilepsy patients [6]. Epilepsy imposes an increase in energy demand not only during initiation and duration of seizures, but also for repair and recovery. Neurometabolic coupling - the relationship between neural activity and energy metabolism in the brain - may be disrupted in epilepsy, leading to alterations in brain function and increased susceptibility to seizures. These findings have been supported by imaging data and physiological measurements in humans and animal models [7]. Most importantly, the strict association between seizure activity, metabolism and cerebrovascular response is the basis of current diagnostic strategies aimed at identifying the zone of seizure onset in many patients as well as in pre-clinical research [8,9].

A key feature of seizures is the increase in energy metabolism that occurs during abnormal neuronal firing, which leads to an increase in glucose metabolism and oxygen consumption. This increased metabolic demand evokes an increase in blood flow, while also producing an accumulation of metabolic intermediates such as lactate, adenosine, and changes in pH [10–15]. Many studies have supported the notion that energy metabolism is compartmentalized between neural cells, with astrocytes presenting a glycolytic profile and neurons an oxidative profile. Furthermore, the glycolytic end product in astrocytes appears to be lactate, which is transported to the extracellular space and then into neurons to fuel the tricarboxylic acid cycle and oxidative phosphorylation [16]. In addition to its role as a non-glucose energy fuel, recent studies have highlighted the role of lactate in the regulation of neuronal excitability.

Evidence for the role of lactate in seizures and epilepsy arises from a set of key observations, including i) lactate-induced reduction of epileptiform activity through HCA1 and G-protein-coupled inwardly rectifying potassium (GIRK) channel activation [17], ii) changes in the expression of monocarboxylate transporters (MCT) in models of epilepsy which may impact the vectorial flux of lactate from astrocytes to neurons [18], and iii) Stiripentol, an anti-epileptic drug, is an inhibitor of lactate dehydrogenase, the enzyme responsible for catalysing the conversion of lactate to pyruvate [19].

*In vivo* electrophysiology and electrochemistry are valuable tools to understand the correlation between different neuronal activities [20]. Regarding epilepsy, electrophysiology can identify the location and timing of seizure activity, while electrochemistry can identify changes in neurotransmitter levels that may be contributing to the seizure [21–26]. Combining the two measurements can provide a more complete picture of the changes that occur in the brain during seizures and how these changes may contribute to the development and spread of epilepsy [27].

The ability of high-frequency amperometry to report both electrochemical and electrophysiological data from a single sensor was initially demonstrated using a choline biosensor, where the authors demonstrated the concurrence between the high-frequency component of the amperometric signal (>1 Hz) and local field potential [28]. This was also verified for biosensors for glucose and lactate [29] as well as oxygen [23]. Other works have also demonstrated that field potentials, effectively field currents, can be reconstructed from the signal of amperometric sensors [30].

In the present work, we aim to extend our previous work on exploring the potential of fast sampling amperometry for concurrent monitoring of chemical and electrophysiological changes associated with seizures. We have combined two carbon fiber microelectrode (CFM)-based biosensors to simultaneously monitor *in vivo* lactate and oxygen during 4-AP-evoked *status epilepticus* in the rat brain with high temporal and spatial resolution. The lactate biosensor was designed by exploring the analytical properties of Pt-nanoparticles and lactate oxidase while the oxygen sensor was based on a multiwall carbon nanotube modified CFM. By using high-frequency amperometry, we are able to extract the local field potential in the form of local field currents and correlate the chemical and electrophysiological changes associated with ictal activity with high spatial overlap. This study provides insights into the role of lactate and oxygen in a rodent model of seizure activity and furthers the development of treatments and therapies for epilepsy.

## 2. Materials and methods

### 2.1. Chemicals and solutions

All chemicals were of analytical grade. Lactate oxidase (LOx) from *Aerococcus viridians* in lyophilized powder form, glutaraldehyde solution (25 %) (GA), bovine serum albumin (BSA), l-lactate sodium salt, hydrogen peroxide solution (30 % w/w), Nafion^®^, hydrogen hexachloroplatinate solution (8 % in H_2_O), polyurethane (PU), tetrahydrofuran (THF), dimethylformamide (DMF), *meta*-phenylenediamine (*m*-PD) and ascorbate were obtained from Sigma Aldrich, Portugal. 4-aminopyridine (4-AP) was obtained from Tocris Bioscience and multiwall CNT (MWCNT) from Nano-lab, USA. All solutions were prepared in bideionized MilliQ water with resistivity ≥18mΩ cm (Millipore Corporation, USA). Argon and oxygen were provided by Air Liquide, Portugal.

The *in vitro* analytical evaluation of microbiosensors was performed in a 0.05 M phosphate-buffered saline (PBS lite) (pH 7.4) with the following composition (mM): 100 NaCl, 10 NaH_2_PO_4_ and 40 Na_2_HPO_4_. The 0.4 % chloroplatinic acid solution was prepared in 0.1 M H_2_SO_4_. Stock solution of lactate (1 M) was conserved at 4 °C protected from light. The working solutions of H_2_O_2_ (9.8 mM) and ascorbate (20 mM) were prepared freshly each day. The solutions used for intracranial pressure-ejections were prepared in saline (0.9 % NaCl) at a pH of 7.4. Saturated O_2_ solutions for calibrations were prepared by bubbling 0.05 M PBS lite with 100 % O_2_ at 37 °C for 20 min, resulting in a 1.04 mM O_2_ solution [31].

### 2.2. Electrochemical instrumentation

Chronoamperometric and voltametric measurements were performed using a MultiPalmSens4 potentiostat controlled by MultiTrace v 4.2 software (PalmSens BV, Houten, The Netherlands) using a three electrode electrochemical cell comprised of a CFM as working electrode, an Ag/AgCl (3 M NaCl) as reference electrode (RE-5B) and Pt wire as an auxiliary electrode. For amperometric calibration and *in vivo* recording of the lactate biosensor and oxygen sensor, a FAST16mkII potentiostat (Quanteon, Nicholasville, KY, USA) was used in a two-electrode electrochemical cell configuration mode comprised of the working electrode and an Ag/AgCl (3 M NaCl) reference electrode. For *in vivo* recordings, an Ag/AgCl wire reference electrode was prepared by electro-oxidation of the exposed tip of a Teflon-coated Ag wire (200 μm o.d., Science Products GmbH, Hofheim, Germany) in 1 M HCl saturated with NaCl, which develops an Ag/AgCl half-cell when in contact with cerebrospinal fluid in the brain containing chloride ions.

### 2.3. Fabrication of carbon fiber microelectrodes

Carbon fiber microelectrodes were fabricated as previously described [32]. Briefly, single carbon fibers (30 μm in diameter) were inserted into borosilicate glass capillaries (1.16 mm i.d. and 2.0 mm o.d., Harvard Apparatus, Holliston, MA, USA) and cleaned with acetone. After drying, each capillary was pulled on a vertical puller (Harvard Apparatus, UK) and the protruding carbon fibers were cut to a tip length of 150–250 μm. Conductive silver paint was used to establish the electrical contact between the carbon fiber and the copper wire. The microelectrodes general recording properties were tested in PBS by fast cyclic voltammetry at a scan rate of 400 V s^−1^, between - 0.4 and +1.6 V *vs*. Ag/AgCl. All CFMs were clean with isopropanol before platinization and MWCNT coating procedures.

#### 2.4. Oxygen sensor fabrication

The oxygen sensor was fabricated by modification of the exposed carbon fiber surface with MWCNT (10 mg ml^−1^ in a 0.5 % Nafion^®^ solution). The MWCNT suspension was placed in an ultrasound bath for 30 min to guarantee the dispersion of the MWCNT and then the CFM tip was dipped in the suspension and stirred slowly for about 30 s and then dried at 170 °C for 5 min (CFM/MWCNT-Nafion^®^) [33].

### 2.5. Lactate biosensor fabrication

The lactate biosensor was prepared as previously described [34–36]. Briefly, the bare CFM was first modified by Pt electrodeposition onto the carbon surface. Potentiostatic electrodeposition was performed by applying a holding potential of - 0.2 V vs. Ag/AgCl for 10 s using a deoxygenated 0.4 % chloroplatinic acid solution in 0.1 M H_2_SO_4_. This was performed in a two-electrode electrochemical cell and using the MultiPalmSens4 Potentiostat (PalmSens, Houten, The Netherlands).

Following the platinization procedure, the Pt-modified CFMs were oven-dried for 5 min at 170 °C to remove traces of humidity. Then, the microelectrodes tips were dipped in a fresh Nafion^®^ solution (5 % in aliphatic alcohols) at room temperature for 5 s and dried in an oven for 15 min at 170 °C. The process was repeated to attain two Nafion^®^ layers. Next, to obtain the lactate microbiosensor (CFM/Pt/Nafion^®^-LOx) the tip of the CFM/Pt/Nafion^®^ was immersed for 5 min in a drop of a cocktail solution containing 5.0 mg mL^−1^ of LOx, 10 % BSA and 0.125 % GA in water. The procedure was repeated three times with 1 min of interval.

Null or sentinel sensors (CFM/Pt/Nafion^®^-Null) for self-referencing recordings were obtained by coating the CFM/Pt/Nafion^®^ tip surface with a protein matrix solution (only BSA and GA), using the same procedure described above.

Both the lactate and null microbiosensors were stored dry at room temperature, protected from light and dust, for at least 72 h for curing. Then, the microsensors were dipped three times in a 2 % solution of PU dissolved in a THF and DMF solution (98 % and 2 % v/v, respectively) with 10 min intervals between dips at room temperature to dry the layers (CFM/Pt/Nafion^®^-LOx/PU; CFM/Pt/Nafion^®^-Null/PU). Finally, on the day of experiments, the sensors surface was modified with an exclusion layer of *m*-PD, to minimize the interference of possible undesirable electroactive compounds. *m*-PD was electropolymerized by CV at a scan rate of 50 mV s^−1^, between +0.25 and +0.75 V *vs*. Ag/AgCl for 20 min (FAST16mkII) using a freshly prepared 5 mM solution of *m*-PD in deoxygenated PBS.

### 2.6. Array assembling

Before brain implantation, the developed lactate biosensor, null sensor and oxygen sensor were assembled with a micropipette into an array. A glass capillary (0.58 mm i.d. and 1.0 mm o.d., Harvard Apparatus, Holliston, MA, USA) was pulled on a vertical puller and the stretched end of the capillary was bumped under a microscope to achieve a opening of 15–20 μm in internal diameter. The lactate biosensor was then attached to the null sensor with the help of wax. Then, the oxygen sensor was attached to the previous ones, followed by the micropipette, using the same procedure. This entire process was made with resource to a microscope to ensure the desire distances between the end points of the biosensor, sensors and micropipette. The final array geometry is depicted in Fig. 3.

### 2.7. In vitro evaluation and characterization

The CFM/MWCNT-Nafion^®^ sensitivity towards oxygen was evaluated by amperometry at - 0.6 V *vs*. Ag/AgCl in 0.05 M PBS lite at 37 °C under moderate stirring. Oxygen was removed by purging the solution with argon for 20 min, after which the needle was removed from the solution and kept above the surface, to decrease O_2_ back-diffusion. When the baseline was stable, four additions of a saturated O_2_ solution were performed (concentration range 5.02–41.6 μM).

The enzyme analytical and kinetic parameters of the CFM/Pt lactate microbiosensors were evaluated by amperometry at + 0.7 V *vs*. Ag/AgCl in 0.05 M PBS at 37 °C, under moderate stirring, by evaluating the response towards consecutive additions of lactate (concentration range: 0.05–20 mM). The sensitivity towards lactate, selectivity against ascorbate, dopamine and uric acid and the sensitivity to the reporter molecule were determined by evaluating the response to three additions of lactate (final concentration range: 0.25–0.75 mM) in the presence of ascorbate 0.25 mM, dopamine 10 μM, and uric acid 20 μM, followed by H_2_O_2_ 10 μM.

### 2.8. Surgical procedures and in vivo experiments

All the procedures used in this study were performed in agreement with the European Union Council Directive for the Care and Use of Laboratory animals (2010/63/EU) and were approved by the local ethics committee (ORBEA). The present *in vivo* studies were carried out on an adult male Wistar rats (8–10 weeks, weight between 250 and 350 g) maintained in the following controlled environmental conditions: temperature of 22–24 °C, relative humidity of 45–65 %, 15 air exchanges per hour, and a 12:12 light/dark cycle; housed in filter-topped type III Makrolon cages on an individually ventilated caging system (VentiRack BioscreenTM, Margate, UK), fed with a standard rat chow diet (4RF21-GLP Mucedola, SRL, Settimo Milanese, Milan, Italy) and provided with chlorinated water, available in *ad libitum*.

Rats were anaesthetized with urethane (1.25–1.50 g/kg, i.p.) and placed in a stereotaxic frame (Stoelting, Wood Dale, IL, USA). Animal temperature was maintained at 37 °C by using a deltaphase isothermal pad (Braintree Scientific, Braintree, MA, USA). The skull was exposed by a midline scalp incision, and then a hole was drilled in the skull over-lying the brain area of interest according to coordinates calculated from bregma based on the atlas of the rat brain [37]. Prior to the insertion of the microbiosensor array into the rat brain, the meninges were refracted. A small hole was drilled in a spot remote from the recording area and a miniature Ag/AgCl pseudo-reference electrode was inserted and soaked with a 0.9 % NaCl solution. The micropipette was filled with a flexible microfilament (MicroFil, World Precision Instruments, Hitchin, UK). Amperometric recordings were started, and the background currents were allowed to stabilize for at least 30 min. After stabilization, solutions were pressure ejected from the glass micropipette using a Picospritzer III (Parker Hannifin Corp., General Value, Fairfield, NJ, USA) into the brain area adjacent to the microbiosensors. To assess the operation stability, the response of the lactate biosensors and oxygen sensors were tested before and after the *in vivo* experiments.

### 2.9. Data analysis

*In vitro* data analysis was performed using GraphPad Prism 8 and OriginPro 2016. For the O_2_ sensor, the sensitivity was determined by the slope of the linear regression analysis between 5.2 and 41.6 μM. Regarding the lactate microbiosensor, enzyme parameters and kinetics were determined with a Michaelis-Menten type equation, with the obtention of the apparent Michaelis-Menten constant for lactate (K_M,app_) and the maximum steady-state current response (*I*_max_). The sensitivity was determined with the slope of the linear regression in the linear range up to 0.5 mM. The selectivity towards interferents was calculated on a molar basis as the ratio of the sensitivity towards lactate and interferent. The limit of detection (LOD) was calculated with the expression LOD = (3×SD)/m where SD is the standard deviation of the baseline and m the slope of the calibration curve. Values are given as the mean ± standard deviation (SD).

Signal processing of *in vivo* recordings was performed using Origin-Pro 2016. The raw signal acquired at 40 Hz was filtered using a low-pass FFT with a cut-off of 1 Hz. The obtained signal was then converted to lactate and oxygen concentration in accordance with the sensitivity obtained in the pre-experiment calibration. The obtained signal was smoothed using a 200-point Savitzky-Golay method. Representing data of the high-frequency component corresponds to the FFT band pass (1–20 Hz) and to the power of the FFT band pass (1–20 Hz) of the raw signal. The frequency power-spectrum was obtained by applying a short time Fourier transform to the high-frequency component, using an FFT length of 256, window length of 64, overlap of 32, and a Triangular window type for the KCl depolarization experiment and an FFT length of 256, window length of 150, overlap of 128, and an Hanning window type for the *status epilepticus* experiment.

## 3. Results and discussion

### 3.1. Analytical performance of the oxygen sensor

**Fig. 1.**
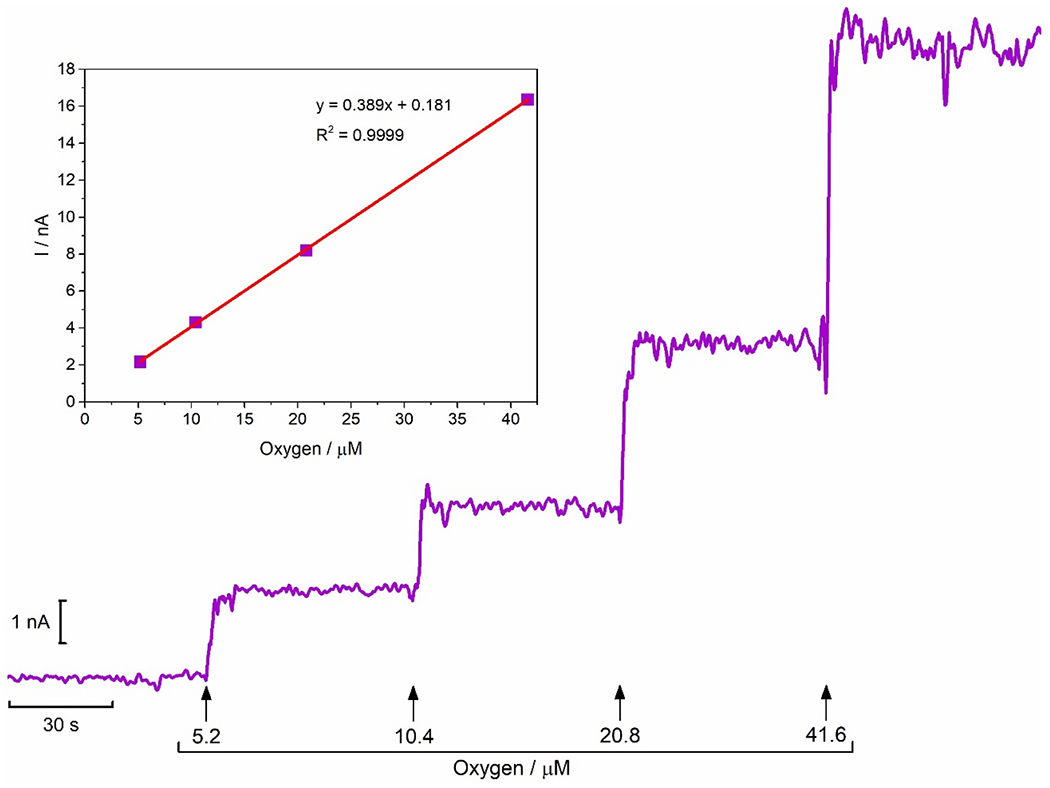
Representative recording calibration of the CFM/MWCNT-Nafion^®^ sensor and the corresponding calibration curve (inset). Calibration was performed by successive additions of a saturated O_2_ solution. The electrode shows a linear response over the concentration range 0–41.6 μM, with a sensitivity of 0.389 nA μM^−1^ and a limit of detection (LOD) of 0.162 μM.

A variety of microelectrodes have been used for the direct reduction of O_2_ including noble metal electrodes (e.g., Pt, Au) and carbon-based electrodes (e.g., carbon paste, carbon fibers, carbon nanotubes) [23, 38–42]. To minimize biofouling resulting from the non-specific adsorption of macromolecules and improve biocompatibility, the surface of the microelectrodes can be coated with BSA, Nafion^®^, PEDOT, Polyurethane and other biocompatible biomaterials [42–46]. Here, we extend our previous work in which we have characterized the electrochemical performance of MEAs for *in vivo* oximetry [23,47], by developing an oxygen microsensor based on a CFM modified with a composite film of MWCNT (10 mg ml^−1^) and Nafion^®^ (0.5 %). The electrochemical response of the oxygen microsensor was evaluated by amperometry at a constant potential of – 0.6 V *vs*. Ag/AgCl, previously reported as the optimal working potential to monitor oxygen [33,42,48]. A typical response to successive additions of O_2_ in the range of 5.2–41.6 μM in N_2_ flushed PBS is depicted in Fig. 1, including the respective calibration curve (inset). On average, the CFM/MWCNT-Nafion^®^ exhibited a sensitivity of - 0.33 ± 0.05 nA μM^−1^ with a linearity of R^2^ = 0.9995 ± 0.0002, a sensitivity/area of −1.79 ± 0.34 mA mM^−1^ cm^−2^, and a LOD of 0.17 ± 0.03 μM (n = 7).

Data are comparable to the O_2_ response of CFM-modified microsensors described by others [42,49–51].

### 3.2. Enzyme kinetics and analytical performance of the lactate biosensor

**Fig. 2.**
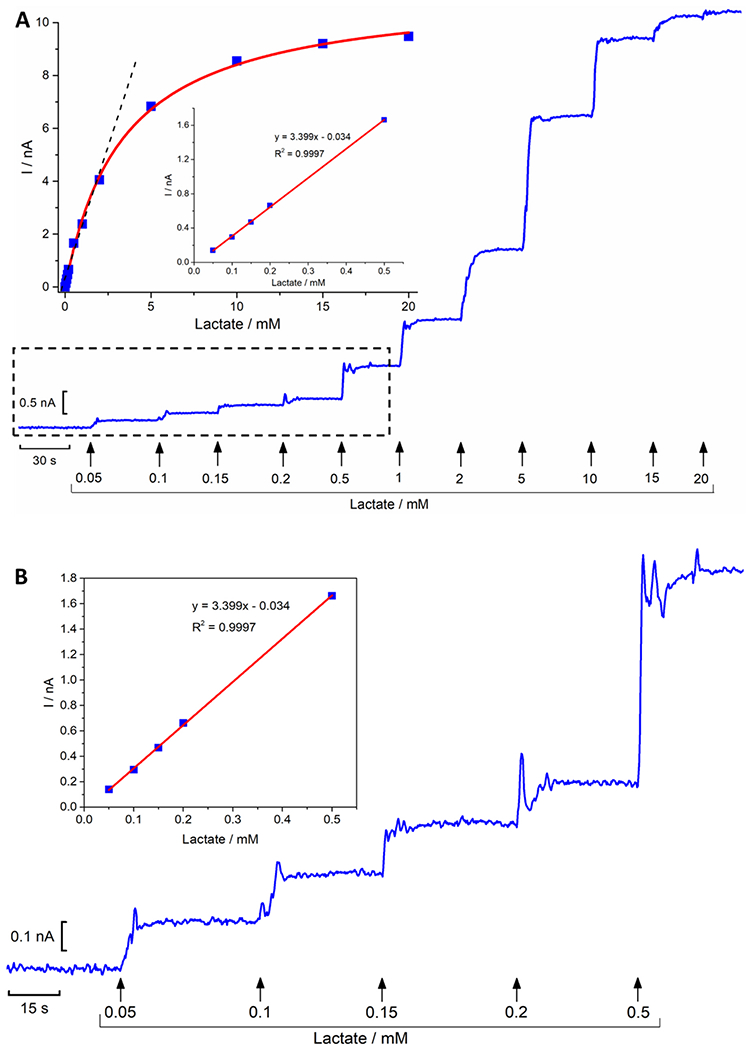
**Panel A:** Representative recording calibration of the CFM/Pt/Nafion^®^-LOx/PU biosensor to successive additions of lactate in the range of 0.05–20 mM. The response was recorded by amperometry at + 0.7 V *vs.* Ag/AgCl. **Inset:** calibration plot of the average steady-state current as function of lactate concentration fitted with a Michaelis-Menten model. **Panel B:** Extended time scale recorded in the lower range of lactate concentration (0.05–0.5 mM). **Inset:** corresponding calibration curve.

The lactate microbiosensors were obtained by immobilizing lactate oxidase (LOx) on the surface of CFM/Pt/Nafion^®^ by cross-linking glutaraldehyde in a BSA matrix followed by coating with PU to extend the linear range of the microbiosensor response. Calibrations with successive additions of lactate were performed to assess the analytical and enzymatic kinetic parameters of the CFM/Pt/Nafion^®^-LOx/PU. A typical response to successive additions of lactate in the range of 0.05–20 mM is shown in Fig. 2. The obtained data was fitted with a simplified one-substrate Michaelis-Menten equation type (inset panel A), giving an average *K*_M,app_ = 2.08 ± 0.86 mM and *I*_max_ = 10.41 ±2.4 nA (n = 5). The lactate microbiosensor displayed a linear response up to 2 mM. Panel B shows the amperometric recording and calibration curve at the lowest lactate concentrations indicated by the dashed box. The average biosensor sensitivity between 0.05 and 0.5 mM towards lactate was found to be 4.27 ± 1.35 nA mM^−1^ (n = 5). The results showed that the lactate microbiosensor exhibited comparable enzyme kinetics and analytical performance to previously described lactate microbiosensors [35].

Given the complex biochemical environment of the brain extracellular space, the selectivity of the lactate biosensor, even with the high specificity of lactate oxidase, is still a critical issue to be improved. Therefore, in addition to coating with Nafion^®^ a well-known negatively charged polymer that repels anions such as ascorbate, both lactate biosensors and null sensors were coated with a permselective membrane of *m*-PD prior to insertion into the tissue. This second layer minimised interference from relevant electroactive compounds such as ascorbate, the main electroactive interferent present in the brain, and resulted in a lactate:ascorbate selectivity of 28.7 ± 1.9:1 (n = 5). The selectivity towards other interferents, such as dopamine and uric acid, was also tested and a selectivity of 6.2 ± 0.3:1 and 4.4 ± 0.9:1 (n = 5) was obtained, respectively.

### 3.3. Concurrent monitoring of electrochemical and electrophysiological data

Concurrent measurements of neurochemicals and LFP with a single sensor probe offer a powerful tool for implantable microelectrode investigating the temporal dynamics of chemical signaling and neural activity and the neural mechanisms underlying behavior and cognition, and for identifying biomarkers of neurological disorders [27,28,30,52–58]. Electrophysiology has proven to be an important tool for understanding epilepsy, due to its ability to directly record neuronal activity with high temporal resolution, allowing the precise identification of seizure onset [59]. Fast sampling amperometry has been shown to be effective for the simultaneous measurements of neurochemicals (*Echem)* and electrophysiological (*Ephys)* signals using a single sensor and recording system [23,27,60]. This is supported by the observation that the high-frequency component of an amperometric recording (greater than 1 Hz) resembles the local field potential (LPF), whereas the low-frequency component (less than 1 Hz) represents the electrochemical signal arising from the oxidation or reduction of electroactive species in the surrounding environment [30,61]. Here, concurrent recordings of *Echem* and *Ephys* were obtained using high-frequency amperometry at a 40 Hz sampling rate. The *Echem* signal was extracted from raw data by applying low-pass filtering up 1 Hz and a high-pass filtering of >1 Hz was applied for LPF.

#### 3.3.1. In vivo monitoring of lactate and oxygen in the brain of anaesthetized rat after KCl depolarization

**Fig. 3.**
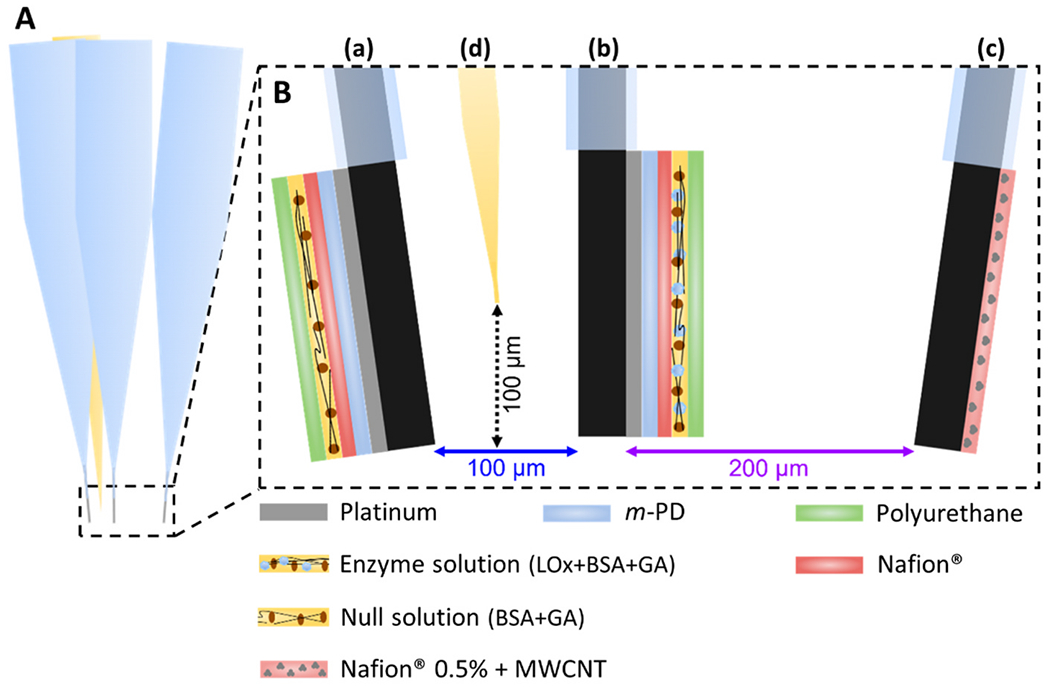
Schematic representation of the microelectrodes and micropipette array. **Panel A:** Side view of the array. **Panel B:** Array tip zone magnification with disclosure of the coating layers of each microelectrode and distances between which microelectrode and micropipette, **(a)** Null sensor; **(b)** Lactate biosensor; **(c)** Oxygen sensor; **(d)** Micropipette.

To evaluate the aptitude of the CFM-based sensors to monitor rapid changes in lactate and oxygen concentrations as well as local field potentials-related currents *in vivo*, we conducted an experiment measuring amperometric currents in the rat hippocampus following high K^+^ stimulation. To accomplish this, we assembled an array containing a lactate biosensor, a null sensor, and an oxygen sensor using a glass micropipette for local solution delivery, as illustrated in Fig. 3.

This sensor array was then inserted into the exposed hippocampus of an anaesthetized rat. After stabilization of the baseline current for all sensors, KCl (70 mM, 500 nL) was pressure ejected from the micropipette.

**Fig. 4.**
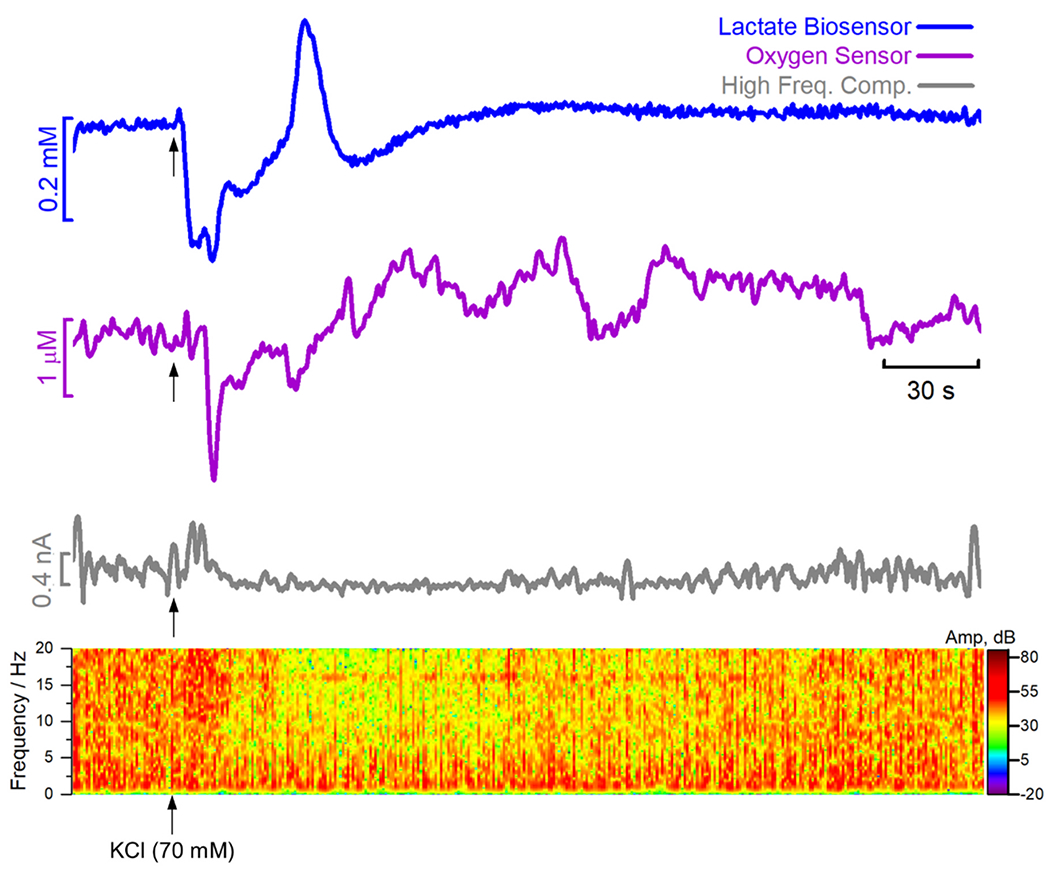
*In vivo* recording of lactate (blue) and oxygen (purple) in the hippocampus of an anaesthetized rat with the CFM/Pt/Nafion^®^-LOx/PU lactate biosensor and the CFM/MWCNT-Nafion^®^ oxygen sensor, respectively. Grey line represents the power of the high-frequency component (1—20 Hz FFT band pass) of the amperometric recording local field potential related currents (LFPrc). Arrow indicates the moment of pressure injection (1 s, 20 Psi) of a potassium chloride solution (70 mM; 500 nL).

As can be seen in Fig. 4, stimulation with KCl led to a rapid and multiphasic fluctuation of extracellular lactate levels in the rat hippocampus (blue trace). An initial decrease in the extracellular concentration of lactate was followed by an overshoot above the baseline which then returned to the baseline levels. The profile of K^+^-evoked change in extracellular O_2_ was also biphasic. However, the initial dip in O_2_ was delayed relative to that of lactate. Also, the overshoot above baseline showed a slower dynamic, most likely reflecting the neurovascular coupling response evoked by K^+^ stimulation. It is interesting to observe that for both lactate and O_2_, the dip in extracellular concentration (reflecting an increase in net consumption of both metabolic substrates) coincides with an initial increase in neuronal activity. This is reflected both in the power spectrogram as well as the mean square of the high frequency component (grey trace) in Fig. 4. Following this, a more prolonged depression in neuronal activity was observed, which then gradually returns to baseline values as the levels of lactate and O_2_ also return to pre-stimulation values. Our results support previous findings indicating that the lactate levels following neuronal stimulation are determined by the dynamic interplay between local glycolytic production and mitochondrial consumption, defining two distinct and sequential phases: an initial dip in lactate levels due to rapid mitochondrial consumption, which is followed by a transient increase when astrocytic production exceeds consumption. Then a decrease when consumption surpasses production [62]. Simultaneous recordings show a high temporal correlation between oxygen and lactate dips and suggest that the transient stimulation of oxidative phosphorylation is followed by a recovery of O_2_ levels by functional hyperaemia [62]. The results highlight the usefulness of such a powerful multiplexed and multimodal approach based on higher spatial and temporal resolution amperometric sensors to simultaneously measure neurometabolic events and ongoing neuronal network activity.

#### 3.3.2. In vivo monitoring of lactate and oxygen during status epilepticus in the brain of anaesthetized rat

In a second study we recorded changes in lactate and O_2_ concurrently with local field potentials during chemically-evoked *status epilepticus* (SE), a condition characterised by hyper-synchronization of neuronal activity and the occurrence of bursts of electrical activity [63–65]. Here we employed an intracortical application of 4-aminopyridine (4-AP) to evoke ictal activity in the rodent cortex. 4-AP is a potassium channel blocker known to induce intense epileptiform activity both in CNS preparations *in vitro* and *in vivo* due to enhanced glutamatergic transmission [66–70]. An array consisting of a lactate biosensor, a null sensor, an O_2_ sensor and a glass micropipette (Fig. 3) was inserted into the cortex of an anaesthetized rat and once stable baselines in all sensors were obtained, SE was evoked by a single pressure ejection of 4-AP (25 mM, 500 nL). It is of notice that the experiments were performed in urethane anaesthetized rodents. Previous studies demonstrated that the use of anaesthetics, namely urethane, may influence the evoked cortical response and local blood flow, which may lead to a lower incidence of seizure activity during experiments [71,72].

**Fig. 5.**
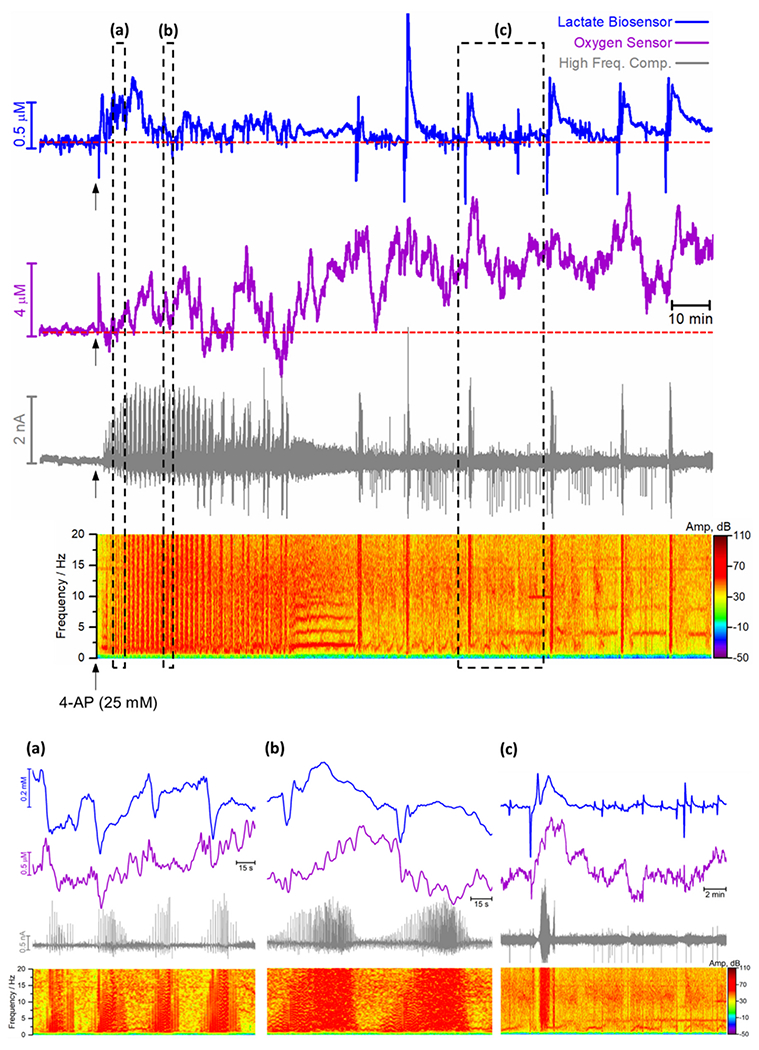
Representative recording of the concurrent measurement of lactate (blue), oxygen (purple) and LFP in the cortex of anaesthetized rat (AP: −4.0; ML: −2.5; DV: −1.5) during *status epilepticus* after 4-AP injection. High frequency component (grey) (1–20 Hz FFT band pass) of the amperometric recording and power spectrum analysis of the high component. Dotted red lines represent the baseline level. Highlighted below are expanded time scales from different moments of the recording (**(a)**, **(b)** and **(c)**).

Fig. 5 shows the temporal correlation between lactate, O_2_ signal and LPFs over an expanded time scale. Following the application of 4-AP, the onset of rhythmic bursting electrical activity (grey trace) was accompanied by an initial transient increase in extracellular lactate (blue trace) which then returns to the baseline value. Inversely, an increase in O_2_ level above baseline (purple trace) was observed. More interestingly, we observed phasic changes in extracellular lactate that were accompanied by O_2_ fluctuations during the different stages of the epileptiform event. As can be observed in the highlights (a), (b) and (c), each individual burst in electrical activity was accompanied by an initial dip in the extracellular lactate concentration followed by an overshoot. Since the measured signal for extracellular lactate reflects the balance between utilization and extrusion, this biphasic response must reflect changes in this balance, with an initial stage in which the increase in electrical activity induces increased consumption of lactate as an oxidative metabolic fuel while the overshoot most likely reflects increased glycolytic rate in astrocytes and lactate release [16,62,73,74].

At this stage, there was a correlation between lactate and O_2_ signals in the ictal or burst phases. However, the transient dip of lactate in the pre-ictal or early burst phase was not accompanied by a significant decrease in O_2_. In the later stages of SE (panels (b) and (c) in Fig. 5), every interictal spike was accompanied by a biphasic change in lactate. This finding provides information on a timescale of milliseconds to a second that cannot be accessible by other techniques, such as brain microdialysis sampling or non-invasive neuroimaging techniques, such as fMRI or PET.

Given the dependence of seizure intensity on specific brain regions and the variability of basal lactate levels in different brain regions [75], it is essential that future studies include other brain regions. Furthermore, experiments in awake/non-anaesthetized rodent models would provide valuable insights.

Epilepsy is now recognised as a disease with a prominent involvement of energy metabolism [6,76]. Our technical approach leveraging the use of microsensor probes for relevant neurometabolic markers combined with amperometry provides a valuable tool for investigating the underlying mechanisms of *status epilepticus* and may thus help the development of novel therapeutic interventions.

## 4. Conclusions

The utilization of amperometric (bio)sensors based on a modified carbon fiber microelectrode platform offers a valuable approach for direct and continuous measurements of neurometabolic markers, including lactate and oxygen in the brain extracellular space. These microsensors exhibit high sensitivity, selectivity, and rapid response time for both neurometabolic markers, enabling *in vivo* measurements with high temporal and spatial resolution.

*In vivo* recordings conducted in the hippocampus of anesthetised rats unveiled rapid and multiphasic fluctuations in extracellular lactate and oxygen levels following neuronal stimulation with high K^+^. This demonstrates the ability of the (bio)sensors to capture rapid dynamic changes in the concentration of neurometabolic markers during neuronal activity. Furthermore, our study employed real-time measurements of lactate and O_2_ concentration dynamics concurrently with network electrical activity during *status epilepticus* induced by 4-AP. The observed phasic changes in lactate levels tightly correlated with bursts of electrical activity, while tonic levels of lactate remained unchanged during seizures. The fluctuations in O_2_ levels above the baseline indicated the involvement of activity-stimulated aerobic glycolysis, which could potentially account for the transient increases in lactate observed during *status epilepticus*.

Overall, our study highlights the interplay between lactate dynamics, electrical activity, and oxygen utilization in seizure-related processes. These findings contribute to a deeper understanding of the metabolic changes occurring during epileptic seizures and pave the way for future research aimed at elucidating the specific mechanisms involved that may uncover novel therapeutic targets for managing *status epilepticus* and/or treatment refractory epilepsy.

## Data Availability

Data will be made available on request.
